# Investigation the Effect of MR Fluid Composition on Properties at Low Strain Ranges

**DOI:** 10.3390/ma16175730

**Published:** 2023-08-22

**Authors:** Anna Fenyk, Wojciech Horak, Marek Zieliński

**Affiliations:** 1 Department of Inorganic and Analytical Chemistry, Faculty of Chemistry, University of Lodz, Tamka 12, 91-403 Lodz, Poland; marek.zielinski@chemia.uni.lodz.pl; 2 Faculty of Mechanical Engineering and Robotics, AGH University of Science and Technology, al. A. Mickiewicza 30, 30-059 Kraków, Poland; horak@agh.edu.pl

**Keywords:** magnetorheological fluids, carbonyl iron, magnetite, magnetic field, DMA, rheology

## Abstract

The paper presents the results of eight magnetorheological (MR) fluids of different compositions. Magnetite and carbonyl iron were used as magnetic particles. MR fluids based on glycerin and OKS 352 oil were produced using stabilizers in the form of oleic acid and Aerosil 200 (Evonik Resource Efficiency GmbH, Hanau, Germany) silica; additives such as graphite and yellow dextrin were also used. The aim of the study was to determine the properties of various combinations of components on the dynamic properties of MR fluids, i.e., properties characterizing the fluid within the range of low deformations, as well as to investigate the effect of different compositions on structural yield stress and flow stress prepared MR fluids at different magnetic field induction values.

## 1. Introduction

Rapid development of science, technology, and industry has resulted in the emergence of a new class of materials often referred to as “smart”. They are characterized by the fact that they can change their properties under the influence of external physical factors, e.g., electric or magnetic fields, mechanical stress, light intensity, temperature, pH etc. These include, among others, magnetorheological (MR) materials, whose rheological and viscoelastic properties, such as the flow limit, shear stress, dynamic modules, and damping properties, can be quickly and reversibly controlled using an external magnetic field [1]. Fluids, elastomers, gels, lubricants, and foams can be distinguished as MR materials [2]. Previous works [3,4,5,6] proved that the factors that affect the rheological properties of MR fluids can be divided into intrinsic, such as the concentration, particle shape and size, the viscosity of the carrier fluid, and the type of additives used. and extrinsic ones, i.e., the value and direction of magnetic field, the shear rate, the temperature. The interdependence of all these factors is very complex and at the same time important when selecting the composition and parameters for a specific application. The flow limit value is one of the main parameters that can be determined from the flow curves [7,8] and which are critical for most applications of MR fluids.

Mineral, silicone, and hydraulic oils, silicone copolymers, polyester, diesters, polyoxyalkylenes, glycols, as well as water are most often used to produce MR fluids [9,10]. In the article by [11], two organic oils (cotton oil and sunflower oil) were used as carrier liquids for the research. Additionally, magnetic particles in the form of carbonyl iron were coated with guar rubber, which ensured better sedimentation stability. It was also found that cottonseed oil-based fluid demonstrates better sedimentation resistance when compared to sunflower oil-based fluid. In the work by [12], the experimental results indicated that ionic liquid carrier-based MR fluids had a higher flow limit and a more significant MR effect than those based on silicone oil with a higher magnetic field induction value. The obtained result was associated with the phenomenon of surface tension of ionic liquids. Since ion fragments are small compared to the size of magnetic particles, they are easily absorbed on their surface, forming an ionic layer. The van der Waals force between them strengthens the interaction between the particles, thus creating a more stable structure. Using silicone oil as a carrier liquid, as in the publication by [13], the MR fluid was prepared. The flow limit was higher in a silicone oil-based fluid when compared to conventional synthetic fluid.

According to Iglesias et al. [14] and Harsh et al. [15], MR fluids consist of magnetizable particles, among others of carbonyl iron, iron oxide, cobalt, nickel, with micron dimensions and a non-magnetizable carrier liquid. They also contain additives affecting their rheological, tribological properties, as well as anti-sedimentation and agglomeration of magnetic particles [16,17,18]. To maximize the MR effect, the base liquid should have low viscosity. In a publication by [19], it was observed that due to the application of an external magnetic field, magnetic particles are aggregated along the direction of the field lines, forming a chain-like structure. The chain structures increase the apparent viscosity of the fluid, causing a change from a liquid state to a solid-like state within milliseconds. The magnetic particles, under the influence of a magnetic field, form chain-shaped structures, which affects the flow limit in the MR fluid [20,21]. In recent years, significant progress has been made in understanding the effect of particle size on the rheological behavior of the material under study [22]. The larger the particles, the greater their magnetization, and therefore the higher the yield stress [23]. A liquid containing colloidal silica and polyethylene glycol mixed with different mass fractions of iron powder was tested, and then the rheological behavior of the obtained substance was analyzed in a publication by [24]. It was observed that the increasing content of carbonyl iron powder effectively increased the viscosity of the fluid under weak magnetic field conditions. According to [25,26], shear stress is an important indicator for the assessment of the rheological properties of liquids, and this important parameter is also influenced by the type of additives used. In the experiment, carbonyl iron particles, two methylsilicone oils, and an additive in the form of stearic acid were used. In a study of the correlation between shear stress and shear rate, it was demonstrated that, under the influence of a magnetic field, the shear stress decreases slightly at the beginning and then gradually increases with a tendency to stabilize, confirming the fact that the larger the magnetic field, the stronger the force of interaction between the chains of particles in the MR fluid, and thus the greater the shear stress. Rheological analysis was carried out on liquids containing carbonyl iron particles, silicone oil, liquid paraffin, graphite, and various additives, including stearic acid, sodium dodecyl sulfate (SDS), and their mixture, as seen in the work of [27]. The results indicated that the rheological properties of MR fluids are affected, in particular, by the mass fraction of magnetic particles. When the mass share of CI was 40–50%, the shear stress first increased and then decreased as the magnetic field strength increased. In contrast, at 60–70% CI mass share, the shear stress of the MR fluid first increased and then stabilized with an increase in magnetic field strength. The authors of [28] presented the results of tests on the rheological properties of magnetorheological fluids with different physicochemical compositions. The tests concerned both the complete compositions of MR fluids and the base liquids (the samples containing no particles with ferromagnetic properties). The paper shows the influence of the selection of individual components of MR fluids on their rheological properties, paying attention to the interaction of the base liquid with particles of the solid phase of the MR fluid. Under magnetic field conditions, the type of ferromagnetic particles (their magnetic properties) have a decisive impact on the MR response of the fluid (change in rheological properties due to the impact of the magnetic field on the fluid).

This article is a more detailed supplement to the work conducted in [28]. The main objective of the work included the assessment of the influence of selected components on one of the key properties of MR fluids, which is the yield stress. The value of this parameter is essential for almost all MR fluid applications, including shock absorbers, brakes, and valves [29,30,31,32]. The problem of determining limit stress values is a complex issue [33,34]. Moreover, in the case of MR fluids, these stresses can be significantly dependent on the deformation conditions, in particular when working in compression mode [35].

The paper focuses on the study of limit stress values of several MR fluids operating in the shear mode, i.e., the mode characteristic of most of the currently known technical applications. It should be noted that the complexity of determining and interpreting limit stresses in MR fluids applies to a large group of liquids with complex composition, in particular non-colloidal suspensions. In such cases, the presence of two characteristic points associated with liquid deformation limit states can be identified. The first of them, referred to as yield stress, corresponds to the value of stress at which the internal structure of the liquid is violated. In this work, the stress corresponding to the linear-elastic range of deformations of the sample was assumed to be this value. In turn, the second characteristic point is flow stress, i.e., the stress value at which the internal structure of the liquid is destroyed. This corresponds to the total flow of the liquid. In the work, the value of tangential stresses corresponding to the equilibrium point of the elasticity and damping moduli was assumed as the flow stress.

Due to the direct relationship of each flow limit with the structural changes occurring in the volume of the MR fluid, the effects of the individual components of the fluid can be expected to be visible in the results of measurements. This paper highlights that individual components can influence the behavior of MR fluids, with differences particularly noticeable at very low deformations. The observed changes can be related to the chemical phenomena described in the paper. It can be also possible to hypothesize that there is a change in the friction coefficient within the fluid. Conclusions about possible changes at the structural level were drawn based on the variability of the measured parameters. If the magnetorheological effect is due to a change in the structure of the fluid, then by observing the rheological quantities it is possible to deduce what is happening inside a suspension.

## 2. Materials and Methods

### 2.1. Preparation of MR Sample

This paper presents the results of investigations into the properties of eight MR fluid samples that differed in composition. The samples were prepared using two types of ferromagnetic particles (magnetite and carbonyl iron), two types of carrier liquid (glycerin and OKS 352 synthetic oil), and two types of stabilizers (oleic acid and Aerosil 200 silica). In addition, Graphite EG 290 and Active carbon and yellow dextrin were added to two samples. All the samples contained a small (0.5%) addition of alumina.

The materials used for preparation of the samples were purchased from commercial sources and used without further purification or chemical treatment. The manufacturers of chemical reagents were: glycerin (Chempur, Piekary Śląskie, Poland), OKS 352 oil (OKS Spezialschmierstoffe GmbH, Maisach—Gernlinden, Germany), Fe_3_O_4_ (magnetite) (Alfa Aesar GmbH, Kandel, Germany), carbonyl iron (Libra, Trzebinia, Poland), oleic acid (Chempur, Piekary Śląskie, Poland), SiO_2_ (Aerosil 200) (Evonik Resource Efficiency GmbH, Hanau, Germany), Al_2_O_3_ (Wolem GmbH, Eschwege, Germany), Graphite EG 290 (3D Nano, Krakow, Poland), Mg(OH)_2_ (Acros Organics, Morris Plains, NJ, USA), activated carbon (Chempur, Piekary Śląskie, Poland), and yellow dextrin (Biomus, Lublin, Poland). Each of the prepared samples contained in its composition respectively: 59 wt.% base liquid, 40 wt.% magnetic particles, 0.5 wt.% each. stabilizer and 5 wt.% of additives, except for samples CM7 and CM8, which contained 54 wt.% base liquid, because they contained that additionally 5 wt.% ingredients in the form of graphite, Mg(OH)_2_ or activated carbon and yellow dextrin. The total was 100% by weight. The procedure consisted in measuring the appropriate amount of a given component and then manually mixing it for about 30 min to obtain a uniform consistency.

The samples produced were marked as CM1 to CM8; the composition of the samples has been presented in detail in Table 1.

The CM7 and CM8 samples contained 54% w/w of the base liquid in their composition due to the fact that they additionally contained 5% w/w of components in the form of graphite, Mg(OH)_2_, or activated carbon and yellow dextrin. The whole constituted 100% mass. Some of the chemical components that are a part of MR fluids can be characterized as follows: OKS 352 oil is a synthetic hydrocarbon oil, resistant to high operating temperatures. SiO_2_ (Aerosil 200) is a matted colloidal silica, with hydrophilic properties, synthetic, amorphous silicon dioxide with a very fine structure, produced by burning silicon tetrachloride in hydrogen furnaces, with 99.8% content of SiO_2_, specific surface area of 200 m^2^/g, bulk density of 0.05 g/cm^3^ and particle diameter of 7–40 nm.

The so-called base liquids were selected for their different properties. OKS 352 synthetic oil protects against oxidation very well, is resistant to high temperatures with optimal wear protection, including under the influence of moisture, prevents corrosion, as well as shows minimal losses due to evaporation (samples C3–C8). Glycerin (C_3_H_8_O_3_) has hygroscopic and hydrophilic properties (samples C1 and C2).

On comparison of the two base liquids with the magnetite (Fe_3_O_4_) content, some differences can be observed. The sample C4 (OKS 352 oil and magnetite) does not change its properties and consistency. The situation is different with the sample C1 (glycerin and magnetite). Glycerin easily absorbs water and is oxidized with oxygen from the air. The magnetite present in the mixture catalyzes the glycerin oxidation reaction, with its decomposition into glyceraldehyde and dihydroxyacetone according to the following Equation (1):
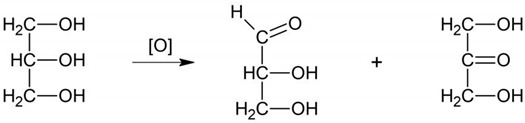
(1)

However, in the environment of glycerin, magnetite itself decomposes into hematite and iron(II) oxide according to Equation (2):

Fe_3_O_4_ →Fe_2_O_3_ + FeO (2)

Hematite under the influence of moisture (water) forms iron(III) hydroxide according to Equation (3):

Fe_2_O_3_ + 3 H_2_O → 2 Fe(OH)_3_ (3)

whereas iron(II) oxide forms iron(II) hydroxide according to Equation (4):

FeO + H_2_O → Fe(OH)_2_ (4)

The sequence of these reactions affects the change in the density of magnetic particles from 5.2 g/cm^3^ to 3.4 g/cm^3^ and the change in the consistency of the whole mixture.

Magnetorheological fluids have been selected so as to contain two different carrier liquids, with different viscosities, two different types of magnetic particles, with different structures, two different types of stabilizers and various additives to change the properties of these liquids, e.g., graphite (EG 290) for thermal conductivity, Mg(OH)_2_ for non-flammable properties, activated carbon to CO_2_ and the other impurities capture properties, or yellow dextrin resulting in biodegradable properties of the liquids.

The graphite used for the CM7 sample is expanded graphite EG 290, with a density of 1.46 g/cm^3^ (bulk density 0.64 g/cm^3^ only) and a granulation of 200–600 μm. The density is identical to that of carbonyl iron with 5–6.5 μm granulation (1.46 g/cm^3^). Expanded graphite (also referred to as intumescent graphite) is graphite modified for the needs of industry. It has a fluffy texture (specific surface area of 100 m^2^/g) and is one of the most delicate minerals.

### 2.2. Characterization of Obtained MRs

**Figure 1:**
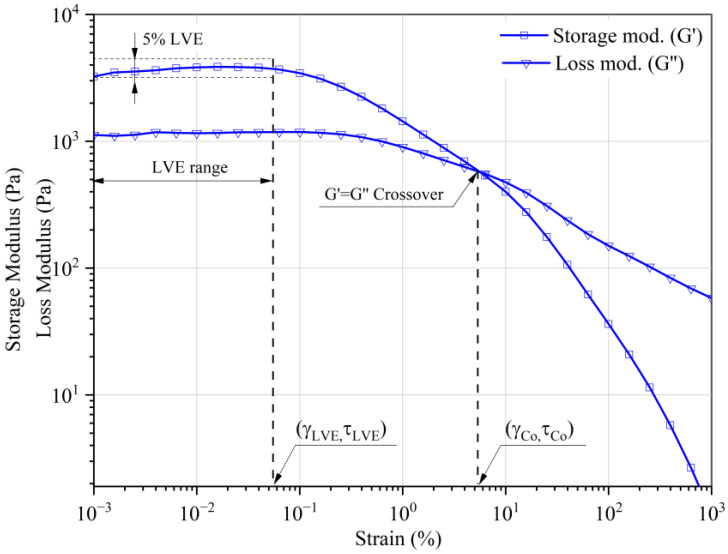
Storage modulus (G′) and loss modulus (G″) vs. strain (γ). Method of determining the yield stress (τ _LVE_) and flow stress (τ _Co_).

The scope of the research included conducting dynamic tests (DMA) to analyze the properties of the produced fluids within the low deformation range, with a specific focus on determining the stress limits (yield stress and flow stress). The scope of research included conducting Amplitude Sweep tests with controlled deformation. In this method, the yield stress is defined as the value of the shear stress at the end of the LVE regime. Here, we define this stress as the point where G′ differs by more than 5% from its strain-independent value in the LVE regime [36,37,38,39]. The flow stress is defined as the value of shear stress at the crossover point where the storage modulus is equal to the loss modulus (G′ = G″) (Figure 1). 

The research was carried out on the Physica MCR 301 (Anton Paar) rotational rheometer, equipped with an MRD-180 magnetic field test cell. The plate-plate geometry (d = 20 mm) was used, the height of the measuring gap (h = 0.5 mm), the volume of the sample (v = 175 μL). The test parameters were as follows: strain (γ = 0.001 to 1000%), changed according to the logarithmic profile (5 points/dec), angular velocity (ω = 101/s), value of the applied magnetic induction (B = 0, 50, 100, 200, 400, and 500 mT). The magnetic field induction values were selected on the basis of previous studies discussed in the paper [28].

The main part of the research was preceded by Frequency Sweep tests, which was the basis for the adoption of a value of angular velocity. The adopted values ensure that tests are carried out both within the scope of controllability of the tested MR fluids, as well as under magnetic saturation conditions. The tests were conducted at t = 25 °C.

## 3. Results and Discussion

### 3.1. Studies of Dynamic Properties of the Examined Mr Fluids

**Figure 2:**
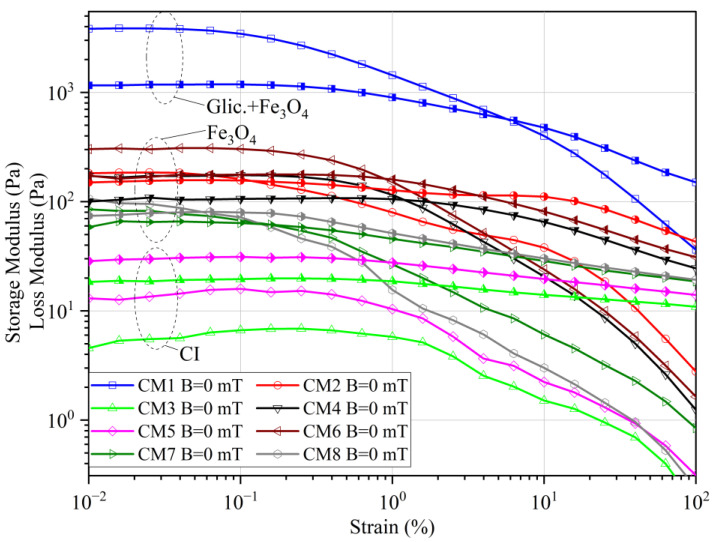
Storage (open symbols) and loss (half empty symbols) modulus at zero state (B = 0 mT).

Figure 2 presents the results of oscillatory tests of produced MR liquids not subjected to magnetic field (in the so-called zero conditions (B = 0 mT)). For all MR fluids tested, a clear linearly elastic range (LVE) was observed. This unexpected behavior of the liquid at zero conditions indicated that all tested MR liquids showed stable elastic properties even before the application of the magnetic field. Under zero conditions, three characteristic groups were distinguished: CM1 liquid, a group of MR liquids containing magnetite and carbonyl iron powder.

CM1 liquid stood out from all tested liquids with high values of elastic moduli (G′ ≈ 3800 Pa), with the second liquid (CM6) characterized by more than 10 times lower elasticity (G′ ≈ 310 Pa).

All liquids assigned to the second group, containing magnetite, have similar properties (G′ ranges from 80 to 310 Pa). As for CM1, there is a clear LVE range and an intersection point of the elastic moduli.

The samples prepared on the basis of carbonyl iron (CI), CM3, and CM5, regardless of additional components, showed the lowest elastic properties (G = 5–12 Pa) among the tested MR fluids. Additionally, only for these two fluids (CM3 and CM5), the value of the loss modulus exceeded the value of the modulus of elasticity, and no intersection point of the curves G′ and G″ was observed. This behavior can be explained by the physicochemical properties of magnetic particles, i.e., carbonyl iron.

**Figure 3:**
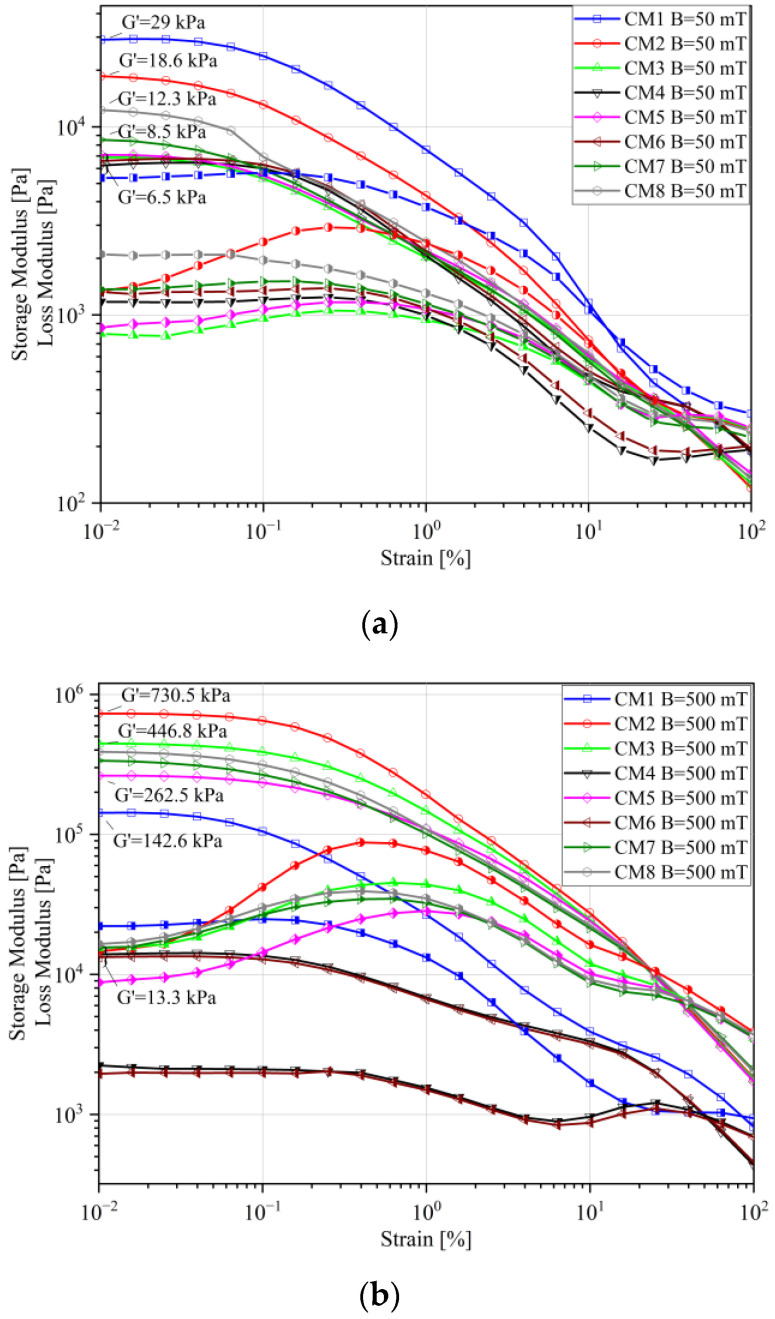
Storage (open symbols) and loss (half empty symbols) modulus: (**a**) B = 50 mT, (**b**) B = 500 mT.

Figure 3 shows examples of results obtained in the presence of a magnetic field. Two ranges were selected: the first one for the lowest analyzed magnetic field induction, i.e., B = 50 mT (Figure 3a), and the second for the highest magnetic induction: B = 500 mT (Figure 3b). In either case, very high values of the elasticity modulus were obtained, which is related to the magnetorheological effect. The value of G′ already at a relatively low magnetic induction was in the order of 6.5–29 kPa, reaching over 730 kPa at B = 500 mT.

Even within the low magnetic induction range (B = 50 mT), the CM1 sample had the highest modulus of elasticity among all tested samples. However, at the highest analyzed magnetic induction value (B = 500 mT), the highest storage modulus values were obtained for the CM2 fluid, i.e., that prepared on the basis of glycerin like CM1, but containing carbonyl iron. In the first case, the high elasticity of the sample in the zero-state turned out to be decisive, while at higher magnetic induction values the effect of the magnetic properties of ferromagnetic particles prevails.

In the context of the loss modulus (G″), a significant intensification of nonlinearity of changes in this parameter is visible with the increase in the value of the applied magnetic field. Greater fluctuations are observed for the samples produced with the use of carbonyl iron (CI).

Differences are noticeable between the CM1 and CM2 samples, considering that the same base liquid was used to produce them, but they differ in the magnetic particles used. These differences, resulting from the physicochemical properties of magnetic particles, may explain the discrepancies in the test results and also affect the form and consistency of the CM1 and CM2 samples. Magnetite is an inorganic chemical compound from the group of oxides with a density of ρ = 5.2 g/cm^3^ and a particle diameter < 45 μm, in which iron is present in the +2 and +3 oxidation states. Smaller fluctuations in the loss modulus (G″) are observed for the samples produced using magnetite (CM4 and CM6). The CM7 fluid differs from other samples containing carbonyl iron. It contains the addition of graphite, which reduced friction between carbonyl iron particles. This was due to the physicochemical properties of the added graphite.

**Figure 4:**
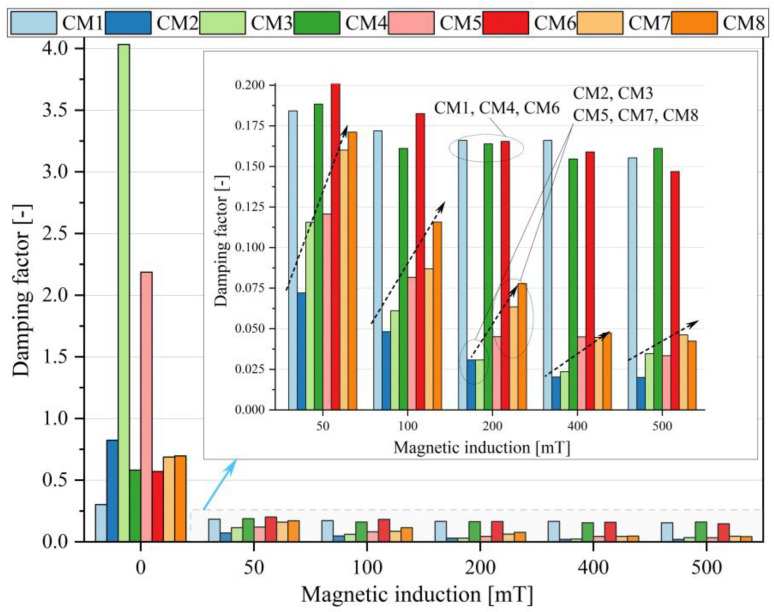
The damping factor at LVE (gamma = 0.01%).

To characterize the dynamic properties of the tested MR fluids, the damping factor tan δ = G″/G′ was determined. For the purposes of comparison, the value of tan δ in the middle LVE range (γ = 0.01%) was recorded; the results are summarized in Figure 4. In the zero state, the CM3 and CM5 samples exhibited the highest damping values, with tan(δ) approximately 4 and just over 2.8, respectively. This indicates a clear advantage of viscous properties over elastic ones. The results suggest that the components used to produce these fluids resulted in the formation of a liquid structure with relatively high internal damping or low elasticity. Notably, only these samples contained carbonyl iron (CI). For the remaining samples, under zero conditions, the values of this parameter were 0.3 for CM1, whereas for samples CM2, CM4, CM6, CM7, CM8, they were similar and fell within the range of 0.6–0.8.

In the case of application of a magnetic field, there is a significant reduction in the damping factor value, which results from the magnetorheological effect and the associated significant increase in the G′ value. The tan(δ) values for all tested samples are within the range of 0.0–0.2. The thumbnail (Figure 4) shows the obtained results on a graph with a scaled ordinate axis. Regardless of the magnetic induction value, the qualitative change in the damping factor is similar for all tested samples. The samples containing magnetite (CM1, CM4, CM6) show relatively high damping, while for other samples (containing carbonyl iron), a trend (marked with arrows) is visible, showing an increase in the damping factor in the individual samples depending on the use of individual additives. The lowest values are visible for the glycerin-based sample (CM2), followed by pure OKS 352 oil (CM3), silica addition (CM5 sample), and graphite and dextrin in the CM7 and CM8 samples, respectively. At the same time, for the highest values of magnetic field induction, the differences in the results between CM7 and CM8 begin to blur.

### 3.2. Studies of the Yield Stresses and Flow Stresses

**Figure 5:**
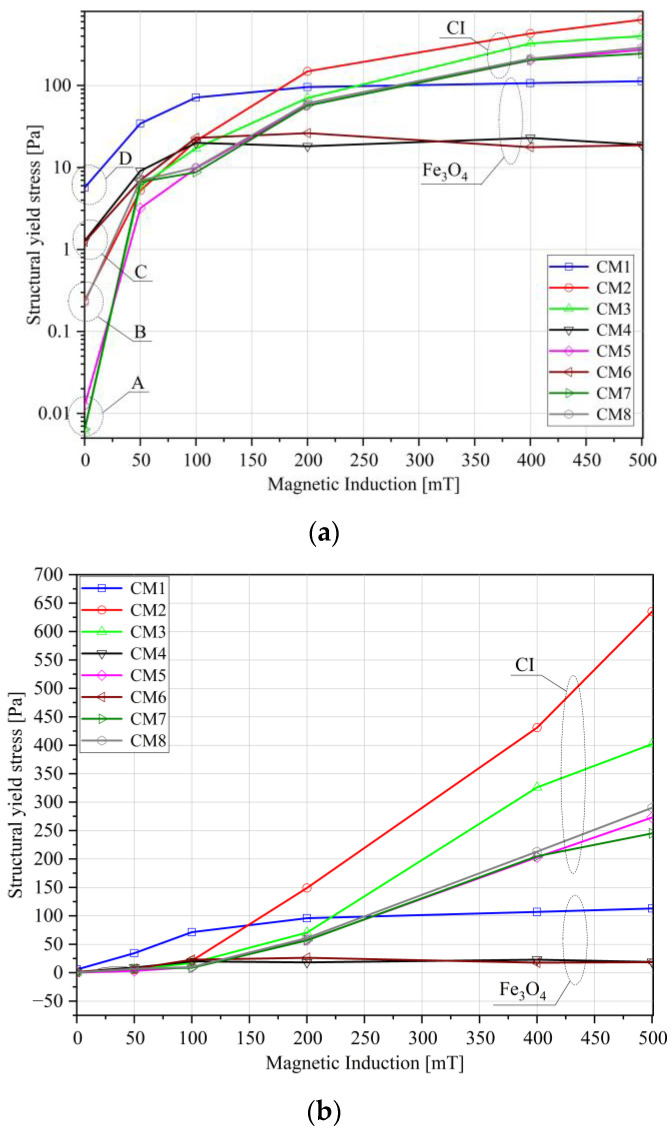
Structural yield stress versus magnetic induction (**a**) in a semi-logarithmic system, (**b**) in a linear system.

Figure 5 illustrates the yield stress as a function of the set value of magnetic field induction. The values were determined to correspond to a 5% change of G′ in the LVE (Linear Viscoelastic) range. To distinguish the range of low magnetic field values, the diagrams are presented in semi-logarithmic (Figure 5a) and linear (Figure 5b) systems.

In the zero state (Figure 5a), a clear division of the tested liquids into four groups is observed: group A, represented by CM5 and CM7 liquids, exhibiting the lowest limit stress values (approximately 0.01 Pa); group B, represented by CM2 and CM3 liquids, showing more than 10× higher values (about 0.11 Pa); group C, represented by CM4 and CM6 liquids, demonstrating another nearly 10-fold increase in stress (to about 1.05 Pa); and the sample with the highest flow stress in the zero state, CM1 (5.7 Pa). Samples from groups A and B were prepared based on carbonyl iron, while groups C and D were based on magnetite.

The samples with magnetite show significantly higher structural yield stress only in the zero state. However, at higher magnetic field induction values (Figure 5b), significantly higher limit stress values are observed for samples with carbonyl iron due to the higher saturation magnetization. There is also a visible saturation of structural yield stress values in the case of liquids produced based on magnetite (CM1, CM4, CM6), beyond which, at B ≈ 100 mT, no further increase in the flow limit is observed.

In the case of carbonyl iron fluids, an increase in shear stress is observed over the entire range of the applied magnetic field induction. There are three groups of liquids: CM2 and CM3 with the highest limit stress values, and CM5, CM7, and CM8, for which similar limit stress values have been obtained. The highest structural yield stress values were obtained for the samples containing the fewest additives. It should be emphasized here that the differences in the value of this parameter are visible only at very low deformations and indicate the effect of the individual components at the level of interactions of the MR fluid molecules.

**Figure 6:**
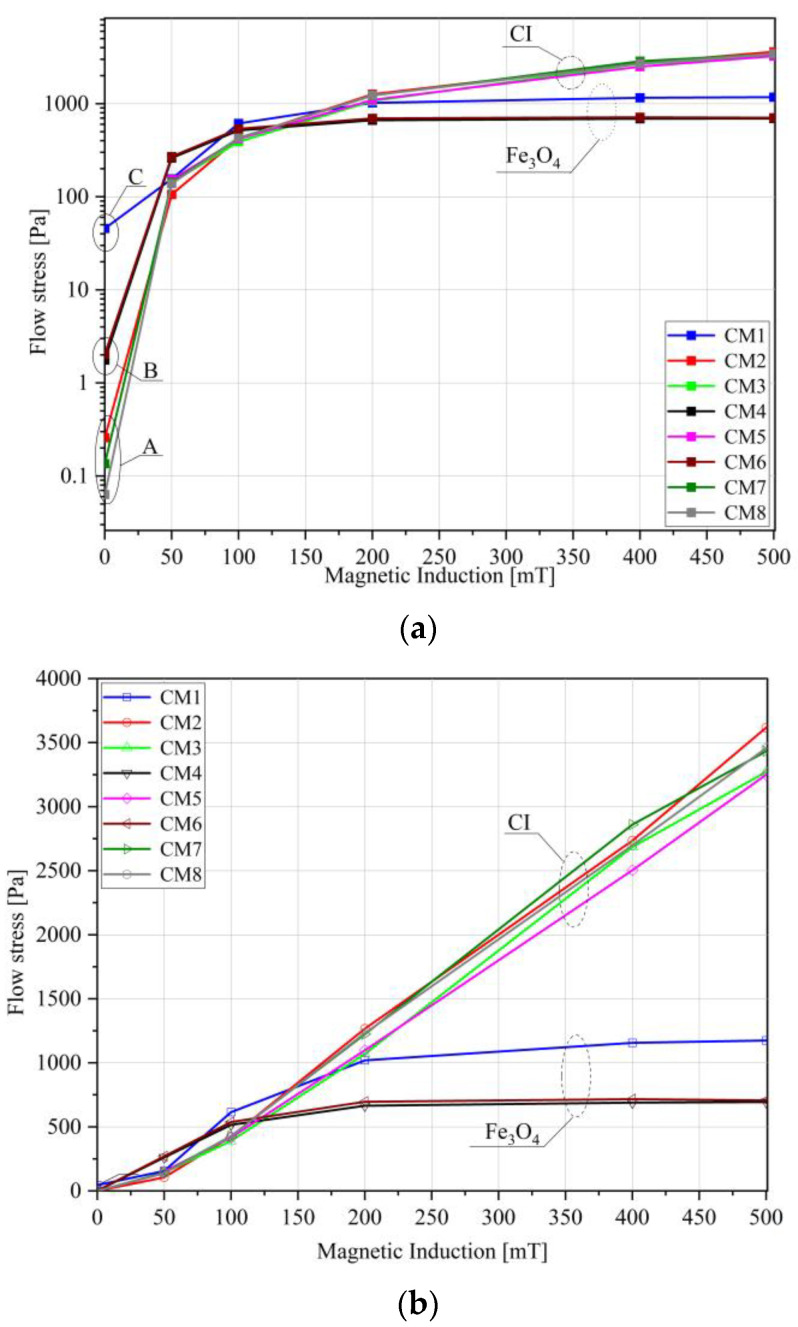
Flow stress versus magnetic induction (**a**) in a semi-logarithmic system, (**b**) in a linear system.

Figure 6 shows the flow stress measurement results. The value of the parameter is determined as the G′ = G″ intersection point. In contrast to structural yield stress, the value of this parameter is determined under the conditions of greater deformation, and its physical sense can be referred to the situation in which the internal structure of the MR fluid is broken.

To better illustrate the results for B = 0 and B ≠ 0, the data are presented on both logarithmic and linear scales. In the zero state, three groups were distinguished: Group A (CM2, CM7, and CM8) with the lowest flow stress (ranging from 0.05–0.2 Pa) (see Figure 6a). The results obtained for CM3 and CM5 also align with the curves of this group, with the difference that no flow stress was recorded for these two liquids under zero conditions. All the Group A fluids were manufactured using carbonyl iron. The other two groups (Group B and Group C) include magnetite-based fluids, and similarly to the structural yield stress, much higher flow stresses were obtained for them in the absence of a magnetic field.

When magnetic induction was applied (Figure 6b), a clear distinction in the samples’ reactions is evident based on the utilized magnetic powder. The higher values of the yield stress were observed for the fluids containing carbonyl iron. This is related to the higher magnetization capability of this material. Therefore, it can be seen that, in the range of low deformations, the influence of the analyzed components on the flow limit is noticeable and can be associated with interactions at the structural level. However, within the range of large deformations, the magnetic properties of ferromagnetic particles come to the forefront.

## 4. Conclusions

The paper presents research results on various MR fluids with differing compositions, types of magnetic particles, viscosity of the carrier liquid, stabilizers, and additives. The study aimed to analyze how the composition affected the dynamic properties and flow limits of the liquids. The differences in physicochemical properties of the magnetic particles explained the discrepancies in test results and influenced the sample’s form and consistency. Dynamic tests (DMA) were conducted to determine the properties of the produced liquids, focusing on structural yield stress and flow stress.

The most significant differences in the behavior of the tested liquids were observed under zero conditions. In the absence of a magnetic field, the rheological behavior of MR fluids depended on the carrier liquid properties and particle properties (number and size). MR fluids with magnetite exhibited higher structural yield stress values under zero conditions. However, at higher magnetic field induction values, samples with carbonyl iron showed much higher stress values due to higher saturation magnetization.

Notably, CM3 and CM5 fluids (differing only in the stabilizer used) exhibited significant differences in behavior only concerning structural yield stress, within the range of very low deformations. Which indicates the apparent impact of the used stabilizer.

The influence of individual additives on rheological properties primarily appeared at low deformations (moduli of elasticity and structural yield stress), indicating their effect near the flow stress. Within the range of higher deformations corresponding to flow stress, differences in the behavior of individual MR fluid samples were much smaller. The main distinctions in the obtained results in this case, were related solely to the type of ferromagnetic powders used.

## Figures and Tables

**Table 1:** Composition of MR fluids samples with the content of magnetic particles.

	Magnetic Powder (40% wt.)	Carrier Fluid	Stabilizer (0.5% wt.)	Addition (0.5% wt.)	Addition (5% wt.)
CM1	Magnetite	glycerine (59% wt.)	Oleic acid	Al_2_O_3_	-
CM2	Carbonyl iron				
CM3	Carbonyl iron	OKS 352 (59% wt.)			
CM4	Magnetite				
CM5	Carbonyl iron		SiO_2_ (Aerosil 200)		
CM6	Magnetite				
CM7	Carbonyl iron	OKS 352 (54% wt.)			Graphite EG 290 Mg(OH)_2_
CM8	Carbonyl iron				Active carbon, yellow dextrin

## Data Availability

Not applicable.
